# Effects of Glutathione S-Transferases *(GSTM1, GSTT1* and *GSTP1*) gene variants in combination with smoking or drinking on cancers: A meta-analysis

**DOI:** 10.1097/MD.0000000000037707

**Published:** 2024-04-05

**Authors:** Qiurui Hu, Cuiping Li, Yonghui Huang, Zhenxia Wei, Li Chen, Ying Luo, Xiaojie Li

**Affiliations:** a College and Hospital of Stomatology, Guangxi Medical University, Nanning, People’s Republic of China; b Guangxi Key Laboratory of Oral and Maxillofacial Rehabilitation and Reconstruction, Guangxi Clinical Research Center for Craniofacial Deformity, Guangxi Health Commission Key Laboratory of Prevention and Treatment for Oral Infectious Diseases, Nanning, People’s Republic of China.

**Keywords:** cancer, drinking, Glutathione S, polymorphism, smoking, transferases

## Abstract

**Background:**

This meta-analysis aimed to systematically summarize the association between cancer risks and glutathione s-transferases (GSTs) among smokers and drinkers.

**Methods:**

Literature was searched through PubMed, Web of Science, CNKI, and WANFANG published from 2001 to 2022. Stata was used with fixed-effect model or random-effect model to calculate pooled odds ratios (ORs) and the 95% confidence interval (95% CI). Sensitivity and heterogeneity calculations were performed, and publication bias was analyzed by Begg and Egger’s test. Regression analysis was performed on the correlated variables about heterogeneity, and the false-positive report probabilities (FPRP) and the Bayesian False Discovery Probability (BFDP) were calculated to assess the confidence of a statistically significant association.

**Results:**

A total of 85 studies were eligible for GSTs and cancer with smoking status (19,604 cases and 23,710 controls), including 14 articles referring to drinking status (4409 cases and 5645 controls). *GSTM1*-null had significant associations with cancer risks (for smokers: OR = 1.347, 95% CI: 1.196–1.516, *P* < .001; for nonsmokers: OR = 1.423, 95% CI: 1.270–1.594, *P* < .001; for drinkers: OR = 1.748, 95% CI: 1.093–2.797, *P = *.02). *GSTT1*-null had significant associations with cancer risks (for smokers: OR = 1.356, 95% CI: 1.114–1.651, *P = *.002; for nonsmokers: OR = 1.103, 95% CI: 1.011–1.204, *P = *.028; for drinkers: OR = 1.423, 95% CI: 1.042–1.942, *P = *.026; for nondrinkers: OR = 1.458, 95% CI: 1.014–2.098, *P = *.042). Negative associations were found between *GSTP1rs1695*(AG + GG/AA) and cancer risks among nondrinkers (OR = 0.840, 95% CI: 0.711–0.985, *P = *.032).

**Conclusions:**

*GSTM1*-null and *GSTT1*-null might be related cancers in combination with smoking or drinking, and *GSTP1rs1695* might be associated with cancers among drinkers.

## 1. Introduction

Cancer is one of the main reasons for the decline of life quality of people all over the world for its high mortality rate, and it brings great challenges to clinical therapy. A former study demonstrated that the number of patients diagnosed as cancer increased by 19.3 million and cases dying from cancer were up to 10 million worldwide in 2020.^[1]^ Lifestyle factors such as smoking and drinking were considered as extremely influential stimulus of the occurrence of cancer. Polycyclic aromatic hydrocarbon, an emission of tobacco, has been regarded as the major organic pollutants affecting human health. A prospective study in 2018 suggested that smokers diagnosed with cancer might be associated with lower survival rate.^[2]^ Some researchers found that long-term smokers and heavy smokers have a significantly higher risk of cancer than the general population.^[3–5]^ Acetaldehyde is the main toxic and harmful substance in the process of alcohol metabolism, which was classified as the group 1 of human carcinogens in the report of International Agency for Research on Cancer. Studies have shown that alcohol consumption increased cancer susceptibility via compromising human immune system and destroying immune mechanism, and this effect was more obvious in Asian populations.^[6,7]^

Glutathione S-transferases (GSTs) supergene family, mainly produced by liver, is one of the most important phase II enzymes in biotransformation in vivo. Each member of GSTs is located on different chromosomes and encoded by one or several highly polymorphic genes.^[8]^ GSTs family plays an essential role in the defense mechanism that protecting against cytotoxic electrophilic chemicals, and GSTs can indirectly control some other metabolizing enzyme’ activities.^[3,9,10]^ Former studies have found that GSTs could reduce the cytotoxic effect by regulating chaperone proteins, ubiquitin-proteasome components, inflammation-related proteins, and apoptosis-related proteins.^[11,12]^ In recent years, many studies have reported that lifestyle factors such as smoking and drinking might lead to changes in enzyme activity levels due to mutations of associated genes. Therefore, it is imperative to explore the mutual regulation and interaction between GSTs polymorphisms and cancers among smoking and drinking population. At present, *GSTM1, GSTT1* and *GSTP1* are the mainstream of research among the members of GSTs. *GSTM1* is located on chromosome 1p13.3, encoding the u class of enzymes. *GSTM1* can detoxifies cellular electrophilic substances by hormonally controlling under induction by phenobarbital and propylthiouracil.^[13]^ *GSTT1* is located on chromosome 22q11.2, encoding for θ class of enzymes. Similar to *GSTM1, GSTT1* can be found in almost all eukaryotes and prokaryotes. The homozygous deletion mutations of *GSTM1* and *GSTT1* might lead to enzyme inactivation and alter the growth activity of certain tumor factors. Due to different coding sites of amino acids, *GSTM1*-null (*GSTM1* -/-) and *GSTT1*-null (*GSTT1* -/-) present the detoxification functional gene deficiency, thus altering susceptibility to some cancers aroused by environmental and lifestyle factors.^[14]^ *GSTP1rs1695*(AA, AG, GG), located on chromosome 11q13, is the most studied gene encoding the π class of enzymes. *GSTP1* polymorphisms were highly associated with alcohol consumption, drug-resistance and the development of cancer.^[15]^ Some studies suggested that cancer risks differs significantly in patients with mutations of GSTs when smoking, drinking, ethnicity and source of controls were taken into account. For example, Katiyar et al, in 2020’ study demonstrated *GSTM1* mutations were associated with a higher incidence of cancer among smokers^[16]^; however, ThekkePurakkal et al, in 2019, explained that there was no statistically significant difference in the effect of *GSTM1* gene polymorphisms on cancer among smokers.^[17]^

Meta-analysis is a robust and scientific statistical analysis method based on huge data, which has incomparable advantages over other research methods, usually having a high credibility. In recent years, the relationship between various cancers and GSTs gene has been studied by scholars worldwide. Xavier et al, in 2017, found that Asian country people with *GSTM1*--null gene were more easily to develop gastric cancer than European and American.^[18]^ Hernández et al^[19]^ in 2017, demonstrated that *GSTM1* and *GSTT1* deletion could not be regarded as a separate factor influencing the survival of lung cancer. Lee et al^[20]^ in 2020, showed that *GSTP1rs1695* polymorphism was useful for the treatment of chronic myeloid leukemia patients. Hoxhaj et al in 2020, found that *GSTM1, GSTT1* and *GSTP1* polymorphisms might increase the risk of developing a second primary cancer among head and neck cancer survivors in different degrees.^[21]^ However, because of the bias of language expression, regions, source and number of cases, there is still lack of a consistent conclusion. Therefore, a large scale of samples and suitable model designs are needed to further evaluate the relationship between GSTs gene and cancer development among smokers and drinkers. The aim of this study was to draw a latest conclusion on the relationship between *GSTM1, GSTT1* and *GSTP1* gene polymorphism and cancer risks among smokers and drinkers. Before us, no similar article has been found to systematically analyze the association between GSTs alone or in combination with smoking or drinking and all kinds of cancers, and we hope that these results will provide some insights into cancer prevention.

## 2. Methods

### 2.1. Literature search and selection criteria

Electronic literatures were searched by using the following databases: Web of Science, PubMed, WANFANG and CNKI. The keywords included (*GSTM1* or *GSTT1* or *GSTP1* polymorphisms) and (smoking or cigarettes or tobacco) or (drinking or alcohol) and cancer by different combinations. The databases were searched in chronological order, from January, 2001 to the latest publication due date November, 2022. Only the case-controls about the association between cancers related to *GSTM1, GSTT1* and *GSTP1* gene polymorphisms among smokers or drinkers that had been published in English or Chinese journals were kept. To reduce omissions as possible, we also searched and consulted references of relevant review articles and meta-analyses. To further investigate the relationship between the degree of smoking and cancer risks, smokers diagnosed with cancers were classified as light smokers (<20 pack-year) and heavy smokers (>20 pack-year).

Articles that confirmed with the following criteria were included: Case-control study; Detailed data on the association of *GSTM1, GSTT1* or *GSTP1* polymorphisms with smoking and alcohol consumption were available for calculating the odds ratios (ORs) and estimating the 95% confidence interval (95% CI); The disease studied was clinically diagnosed cancer; and Full text available.

The reasons for exclusion were as follows: Review articles and meta-analyses as well as repeated articles in different databases; Minutes of meeting and clinical trials; No detailed data of case group and control group; and No original data on the association of *GSTM1, GSTT1* or *GSTP1* gene polymorphisms with smoking or drinking status.

### 2.2. Data extraction

After screening all articles according to the exclusion and inclusion criteria, we performed detailed data extraction for the articles including first author’s last name, year of publication, ethnicity, country, source of control, cancer type, smoking or drinking status and genotype. For case-control studies on the same cancer published by the same author in different years, we kept the latest articles or the maximum sample size in principle. Two researchers used the same keywords to search articles independently. When an article contained unextractable data or some doubts, the 2 researchers would discuss together whether to keep this article. Subgroup analyses were conducted on the ethnicity (Caucasian, Asian, and mixed groups), the source of control group (hospital-based group and population-based group), and the types of cancer (lung cancer, liver cancer, bladder cancer, and so on) to calculate the differences in the prevalence of cancers, respectively.

### 2.3. Statistical analysis

The Stata software was applied to calculate the *GSTM1*-null/presence, *GSTT1* -null/presence, and *GSTP1*rs1695 GG + AG/AA of the case group and control group among different smoking and drinking status. OR value and 95% CI were calculated to evaluate the association between *GSTM1, GSTT1* and *GSTP1* polymorphisms and cancer risks among smokers and drinkers. *I*-square and *P* value were used for the assessment of heterogeneity. If the I-square of the heterogeneity test was less than 50% (*P *> .05), it showed that the heterogeneity between studies was not statistically significant, and the fixed model should be used for calculation; otherwise, the random model would be applied.

In this study, year, ethnicity, and population origin were considered to be the sources that could influence heterogeneity, and the meta-regression analysis were used to find these variables. At the same time, combined with the use of sensitivity analysis, any study that had an impact on the overall results of the study could be identified. We also used the Begg and Egger’s test to calculate possible bias between studies. All *P* values calculations were two-sided, and when *P* value < .05, it was considered to be statistically significant.

In order to improve the accuracy and credibility of the results of this experiment, we calculated the false positive reporting probability (FPRP) and the Bayesian false discovery probability (BFDP). As in previous studies, the threshold of FPRP was set to 0.2, and the prior probability was set to 0.25, 0.1, 0.01, 0.001 and 0.0001, to detect an OR of 1.5 associated with cancer risks in the study; and results with FPRP values less than 0.2 should be of concern.^[22]^ Likewise, the BFDP results should be noted when the *P* value was less than 0.8.^[23]^

## 3. Results

### 3.1. Study characteristics

**Figure 1.**
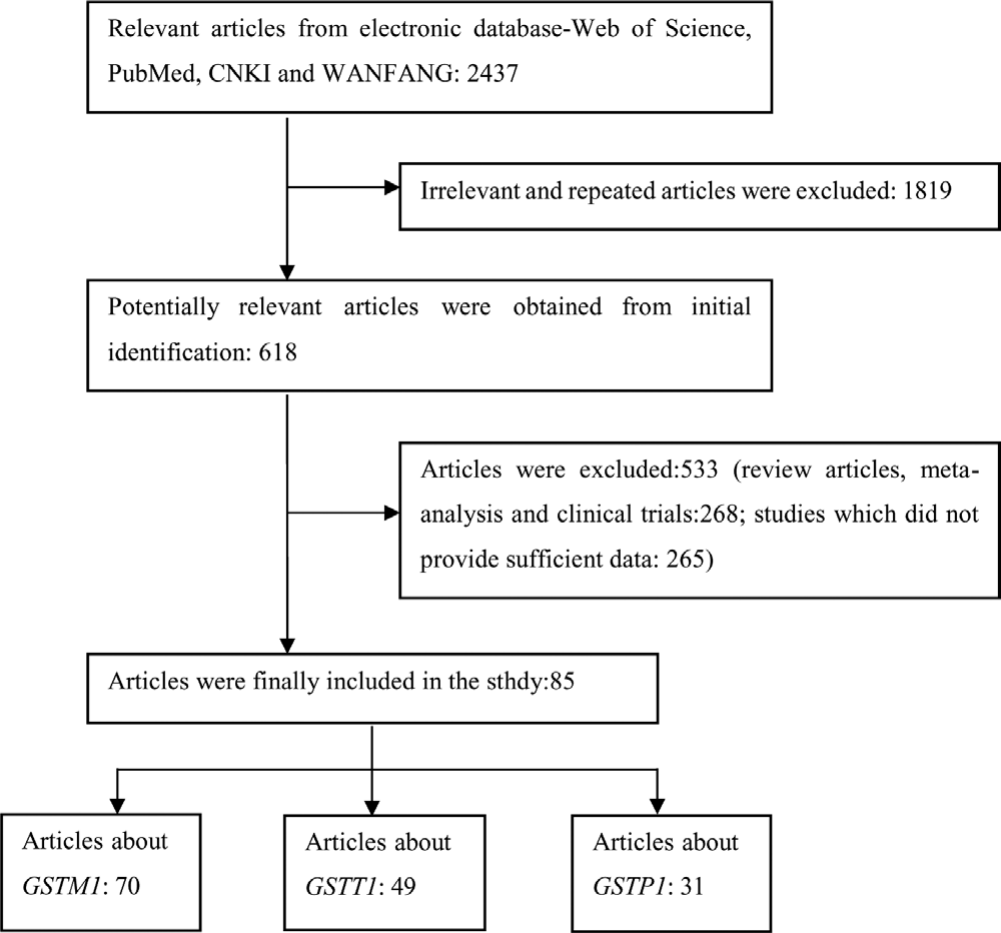
The whole flow diagram of filtering the available articles in this study.

By using keywords, a total of 2437 related reports were found in digital databases. After scanning the titles and abstracts firstly, 2086 articles were excluded, including reviews, meta-analyses, clinical trials, irrelevant reports and duplicate reports. By carefully reading the whole text of remaining studies, we further removed 266 articles for the following reasons: lack of the information for number of samples; lack of research content on the association between genetic polymorphisms and cancers among smoking or drinking population. Finally, 85 articles (19,604 cases and 23,710 controls) were kept (Fig. 1), of which 70 articles (16,131 cases and 19,696 controls) were about the relationship between *GSTM1* and cancers^[4,5,16,17,24–89]^ (Table 1); 49 articles (11,555 cases and 14,606 controls) were about the relationship between *GSTT1* and cancers^[4,5,16,24,25,27–31,33,34,36–39,41,42,44–49,51,52,56,58,60,61,63,65–68,70,74–76,79,80,82–85,87,88,90–93]^ (Table 2); and 31 articles (8518 cases and 9884 controls) were about *GSTP1* and cancers^[5,16,17,24,30,31,34,36,42,44,45,47,49,52,59,66,67,70,79,88,94–103]^ (Table 3) among smokers. Among these researches, 8 articles mentioned the classification of smoking levels (20 pack-year). For alcohol consumption, 12 articles (4238 cases and 5394 controls) were about the association between *GSTM1* and cancers, 8 articles (2949 cases and 4025 controls) were about *GSTT1* and cancers and 5 articles (1898 cases and 2527 controls) were about *GSTP1* and cancer among drinkers.

**Table 1:** Characteristics of the eligible studies for *GSTM1* polymorphisms.

Author	Year	Ethnicity	Cancer type	Source of control	Case (N)	Control (N)	Case (present/null)				Control (present/null)			
							Smoking	Nonsmoking	Drinking	Non-drinking	Smoking	Nonsmoking	Drinking	Non-drinking
Firigato^[89]^	2022	Mixed	Head and neck cancer	HB	234	422	205/16	13/1			272/31	94/17		
Chorfi^[25]^	2022	Caucasian	Liver cancer	NA	132	141	29/58	19/26			36/38	37/32		
Deek^[24]^	2021	Mixed	Lung cancer	NA	200	200	42/60	75/23			59/40	94/7		
Avirmed^[26]^	2021	Asian	Bladder cancer	HB	60	60	19/24	6/11			2/12	15/31		
Pathak^[27]^	2021	Caucasian	Lung cancer	NA	237	212	109/26	69/28			82/11	106/13		
Tcheandjieu^[28]^	2020	Mixed	Thyroid cancer	PB	660	734	116/153	133/213	173/236	76/130	134/168	163/207	222/290	75/85
Katiyar^[16]^	2020	Caucasian	Head and neck cancer	HB	1250	1250	421/424	260/145	165/274	516/295	262/105	603/280	205/119	660/266
Ritambhara^[34]^	2019	Caucasian	Lung cancer	HB	120	100	30/82	4/4			11/15	54/20		
Singh^[29]^	2019	Caucasian	Nasopharyngeal cancer	PB	123	189	23/37	18/45			31/15	76/67		
Rostami^[30]^	2019	Caucasian	Chronic myeloid leukemia	NA	104	104	19/38	15/32			17/14	36/37		
Yamashita^[31]^	2019	Asian	Hypopharyngeal cancer	NA	61	71	22/8	22/9			6/7	41/17		
Thekkepaurakkal^[17]^	2019	Caucasian	Head and neck cancer	HB	389	429	151/170	27/60			142/169	51/65		
Li^[32]^	2019	Asian	Lung cancer	NA	217	198	49/71	41/56			40/23	64/71		
Kalacas^[33]^	2019	Asian	Breast cancer	NA	136	136	6/6	52/72	8/10	50/68	4/7	39/86	8/22	35/71
He^[35]^	2018	Asian	Lung cancer	PB	313	330	66/48	113/86	87/41	122/93	50/28	167/85	61/36	156/77
Rodrigues-Fleming^[36]^	2018	Caucasian	Colorectal cancer	NA	232	738	48/54	52/78	44/56	56/76	154/119	231/234	179/164	206/189
Peddireddy^[37]^	2016	Caucasian	Lung cancer	PB	246	250	106/42	76/22			54/21	133/42		
Boccia^[38]^	2015	Caucasian	Liver cancer	HB	221	290	62/57	31/48			48/69	91/81		
Sharma^[39]^	2015	Caucasian	Cervical cancer	HB	135	482	9/13	47/66			26/17	290/153		
Maurya^[40]^	2015	Caucasian	Head and neck cancer	PB	750	750	105/103	64/28	29/96	140/40	159/61	356/173	141/89	374/145
Pan^[41]^	2014	Asian	Lung cancer	PB	523	523	104/135	114/170			137/102	162/122		
Silva^[42]^	2014	Mixed	Upper aerodigestive tract cancer	PB	116	224	47/42	4/2			54/34	69/45		
Wang^[43]^	2014	Asian	Bladder cancer	NA	358	434	73/124	61/100			75/103	106/150		
Jiang^[44]^	2014	Asian	Lung cancer	NA	322	456	65/151	48/58			115/75	130/136		
Yamada^[45]^	2014	Asian	Pancreatic cancer	PB	360	400	44/52	67/78			23/35	98/104		
Stosic^[46]^	2014	Caucasian	Cervical cancer	HB	97	50	13/39	12/33			11/16	11/12		
Matic^[47]^	2013	Caucasian	Bladder cancer	HB	201	122	74/75	16/36			39/27	21/34		
Lu^[50]^	2013	Asian	Lung cancer	HB	91	138	22/33	8/28			30/32	38/38		
Shukla^[48]^	2013	Caucasian	Lung cancer	HB	218	238	102/47	32/37			42/26	106/64		
Berber^[49]^	2013	Caucasian	Prostate cancer	NA	115	115	31/34	23/27			27/27	32/29		
Kang^[51]^	2013	Asian	Bladder cancer	NA	110	220	34/37	11/28			63/36	54/67		
García-González^[52]^	2012	Caucasian	Gastric cancer	HB	557	557	51/71	125/120			40/35	151/147		
Yao^[53]^	2012	Asian	Lung cancer	HB	150	150	38/67	16/29			42/30	40/38		
Chen^[54]^	2012	Asian	Lung cancer	HB	200	200	57/89	20/34			50/63	29/47		
Zhang^[55]^	2012	Asian	Nasopharyngeal cancer	NA	45	30	8/12	10/15			10/3	9/8		
Liu^[56]^	2012	Asian	Lung cancer	NA	360	360	152/103	63/42			130/55	123/52		
Rouissi^[61]^	2011	Caucasian	Bladder cancer	NA	125	125	39/50	11/9			31/27	38/29		
Li^[57]^	2011	Asian	Lung cancer	NA	103	138	37/43	7/20			41/33	36/28		
Du^[58]^	2011	Asian	Lung cancer	HB	125	125	35/41	17/32			20/23	36/46		
Ramzy^[59]^	2011	Caucasian	Lung cancer	NA	48	42	27/6	6/9			13/1	15/13		
Karageorgi^[60]^	2011	Caucasian	Cervical cancer	NA	137	411	69/83	74/92			233/280	191/207		
Zheng^[62]^	2010	Asian	Liver cancer	HB	266	307	65/108	49/36			63/78	68/96		
Fan^[63]^	2010	Asian	Lung cancer	HB	58	60	9/17	9/23			9/11	18/22		
Jin^[64]^	2010	Asian	Lung cancer	NA	150	150	43/70	12/25			36/51	35/28		
Souiden^[68]^	2010	Caucasian	Prostate cancer	PB	110	122	39/43	13/15			43/54	11/14		
Asim^[65]^	2010	Caucasian	Liver cancer	PB	254	525	84/117	18/35	15/128	87/24	244/128	124/29	101/114	267/43
Li^[66]^	2010	Mixed	Oesophageal cancer	HB	245	288	151/26	55/8	143/22	63/12	125/60	75/20	123/44	77/36
Palma^[67]^	2010	Caucasian	Cervical cancer	PB	81	111	7/21	18/16			11/21	24/21		
Huang^[69]^	2009	Asian	Gastric cancer	HB	121	138	30/27	25/39	27/28	28/38	28/8	56/46	28/11	58/43
Altayli^[70]^	2009	Caucasian	Bladder cancer	PB	135	128	55/39	20/15			25/38	24/23		
Li^[71]^	2008	Asian	Esophageal carcinoma	HB	125	125	43/60	5/8			54/81	16/12		
He^[72]^	2008	Asian	Liver cancer	HB	105	151	20/35	17/33	27/37	10/31	33/27	41/50	44/26	30/51
Chen^[73]^	2008	Asian	Lung cancer	HB	158	455	46/73	13/26			88/120	120/126		
Xie^[74]^	2008	Asian	Gastric cancer	HB	70	100	12/18	13/27	14/15	17/24	20/9	41/30	25/8	36/31
Boccia^[75]^	2007	Caucasian	Gastric cancer	PB	102	254	24/26	24/33	30/43	16/16	44/64	76/70	63/70	57/64
Agorastos^[76]^	2007	Caucasian	Cervical cancer	PB	176	114	41/45	19/43			20/30	19/30		
Qian^[77]^	2006	Asian	Lung cancer	NA	108	108	31/54	8/15			31/31	24/22		
Wang^[78]^	2006	Asian	Lung cancer	NA	56	42	7/30	9/10			11/12	12/7		
Huang^[4]^	2006	Mixed	Colon cancer	NA	554	874	184/158	111/97			292/219	211/151		
Peters^[79]^	2006	Caucasian	Head and neck cancer	NA	692	753	231/319	56/84			228/236	117/168		
Shao^[80]^	2006	Asian	Bladder cancer	NA	414	404	88/178	49/110			96/115	73/105		
Qiao^[81]^	2005	Asian	Lung cancer	HB	213	199	67/108	17/21			63/55	41/40		
Tamer^[5]^	2005	Caucasian	Gastric cancer	HB	70	204	13/21	17/19			41/43	75/45		
Gelatti^[82]^	2005	Caucasian	Liver cancer	HB	200	400	60/65	41/34			125/135	68/80		
Alexandrie^[83]^	2004	Caucasian	Lung cancer	PB	524	530	102/94	13/18			130/142	99/133		
Ravina^[84]^	2003	Caucasian	Lung cancer	HB	132	192	52/63	1/9			58/52	42/35		
Risch^[85]^	2003	Caucasian	Laryngeal cancer	NA	245	251	113/123	5/4			81/102	35/33		
Wang^[86]^	2002	Asian	Esophageal carcinoma	HB	127	101	41/28	33/25			18/20	26/37		
Zheng^[87]^	2002	Caucasian	Breast cancer	NA	338	345	81/103	66/62			83/97	63/76		
Toruner^[88]^	2001	Caucasian	Bladder cancer	PB	121	121	27/53	12/19			34/33	21/17		

Study groups with more than one race or not explicitly mentioned.

HB = hospital-based group, NA = not available, PB = population-based group.

**Table 2:** Characteristics of the eligible studies for *GSTT1* polymorphisms.

Author	Year	Ethnicity	Cancer type	Source of control	Case (N)	Control (N)	Case (present/null)				Control (present/null)			
							Smoking	Nonsmoking	Drinking	Non-drinking	Smoking	Nonsmoking	Drinking	Non-drinking
Chorfi^[25]^	2022	Caucasian	Liver cancer	NA	132	141	60/27	33/12			41/31	50/19		
Deek^[24]^	2021	Mixed	Lung cancer	NA	200	200	80/55	44/21			68/22	80/30		
Pathak^[27]^	2021	Caucasian	Lung cancer	NA	237	212	105/24	64/27			96/11	73/14		
Tcheandjieu^[28]^	2020	Mixed	Thyroid cancer	PB	660	734	206/63	248/77	316/89	138/51	218/76	271/75	373/118	116/33
Katiyar^[16]^	2020	Caucasian	Head and neck cancer	HB	1250	1250	606/239	327/78	295/144	638/173	290/77	715/168	253/71	752/174
Ritambhara^[34]^	2019	Caucasian	Lung cancer	HB	120	100	22/90	6/2			14/12	59/15		
Singh^[29]^	2019	Caucasian	Nasopharyngeal cancer	PB	123	189	38/22	29/34			31/15	89/54		
Rostami^[30]^	2019	Caucasian	Chronic myeloid leukemia	NA	104	104	55/2	46/1			30/1	73/0		
Yamashita^[31]^	2019	Asian	Hypopharyngeal cancer	NA	61	71	20/10	20/11			7/6	36/22		
Kalacas^[33]^	2019	Asian	Breast cancer	NA	136	136	10/2	65/59	8/10	67/51	9/2	68/57	18/12	59/47
Rodrigues-Fleming^[36]^	2018	Caucasian	Colorectal cancer	NA	232	738	82/20	110/20	84/16	108/24	211/62	362/103	273/70	300/95
Peddireddy^[37]^	2016	Caucasian	Lung cancer	PB	246	250	111/37	89/9			66/9	158/17		
Boccia^[38]^	2015	Caucasian	Liver cancer	HB	221	290	81/38	59/20			91/26	129/43		
Sharma^[39]^	2015	Caucasian	Cervical cancer	HB	135	482	14/8	95/18			33/10	380/59		
Pan^[41]^	2014	Asian	Lung cancer	PB	523	523	117/122	136/148			144/99	171/113		
Silva^[42]^	2014	Mixed	Upper aerodigestive tract cancer	PB	116	224	78/11	5/1			70/18	84/30		
Jiang^[44]^	2014	Asian	Lung cancer	NA	322	456	109/90	73/50			131/94	120/111		
Yamada^[45]^	2014	Asian	Pancreatic cancer	PB	360	400	47/49	71/74			28/30	103/99		
Stosic_46_	2014	Caucasian	Cervical cancer	HB	97	50	30/22	29/16			18/9	12/11		
Shi^[90]^	2014	Asian	Gastric cancer	NA	60	83	10/29	16/5			18/11	32/22		
Matic^[47]^	2013	Caucasian	Bladder cancer	HB	201	122	104/45	40/12			48/18	40/16		
Shukla^[48]^	2013	Caucasian	Lung cancer	HB	218	238	85/64	51/18			53/15	127/43		
Berber^[49]^	2013	Caucasian	Prostate cancer	NA	115	115	49/16	39/11			49/5	50/11		
Kang^[51]^	2013	Asian	Bladder cancer	NA	110	220	32/39	14/25			36/63	56/65		
García-González^[52]^	2012	Caucasian	Gastric cancer	HB	557	557	97/25	188/57			56/19	228/70		
Liu^[56]^	2012	Asian	Lung cancer	NA	100	135	18/31	25/26			32/18	47/38		
Rouissi^[61]^	2011	Caucasian	Bladder cancer	NA	125	125	71/18	13/7			38/20	49/18		
Du^[58]^	2011	Asian	Lung cancer	HB	125	125	43/33	22/27			25/18	45/37		
Karageorgi^[60]^	2011	Caucasian	Cervical cancer	NA	137	411	124/24	144/21			406/108	322/74		
Bai^[91]^	2011	Asian	Lung cancer	HB	106	250	32/30	24/20			76/40	63/71		
Fan^[63]^	2010	Asian	Lung cancer	HB	58	60	8/18	12/20			11/8	20/21		
Souiden^[68]^	2010	Caucasian	Prostate cancer	PB	110	122	58/24	22/6			84/13	20/5		
Asim^[65]^	2010	Caucasian	Liver cancer	PB	254	525	120/81	36/17	67/76	89/22	305/67	132/21	143/72	294/16
Li^[66]^	2010	Mixed	Oesophageal cancer	HB	245	288	100/77	27/36	89/76	38/37	112/73	66/29	101/66	77/36
Palma^[67]^	2010	Caucasian	Cervical cancer	PB	81	111	21/7	24/10			26/6	35/10		
Zhang^[92]^	2010	Asian	Pancreatic cancer	HB	150	150	33/64	13/40			54/7	29/60		
Altayli^[70]^	2009	Caucasian	Bladder cancer	PB	135	128	73/21	27/8			60/3	43/4		
Xie^[74]^	2008	Asian	Gastric cancer	HB	70	100	7/23	15/25	7/22	15/16	15/14	35/36	15/18	35/32
Boccia^[75]^	2007	Caucasian	Gastric cancer	PB	102	254	32/18	36/21	47/26	19/13	85/23	112/34	100/33	97/24
Agorastos^[76]^	2007	Caucasian	Cervical cancer	PB	176	114	45/41	41/21			32/18	22/27		
Huang^[4]^	2006	Mixed	Colon cancer	NA	554	874	259/83	162/46			356/155	267/115		
Peters^[79]^	2006	Caucasian	Head and neck cancer	NA	692	753	452/98	116/24			357/108	231/54		
Shao^[80]^	2006	Asian	Bladder cancer	NA	414	404	134/132	67/72			106/105	88/90		
Tamer^[5]^	2005	Caucasian	Gastric cancer	HB	70	204	24/10	25/11			66/18	85/35		
Gelatti^[82]^	2005	Caucasian	Liver cancer	HB	200	400	101/24	67/8			204/56	124/16		
Alexandrie^[83]^	2004	Caucasian	Lung cancer	PB	524	530	231/24	26/5			233/142	200/32		
Ravina^[84]^	2003	Caucasian	Lung cancer	HB	132	192	91/24	7/3			85/25	56/21		
Zheng^[87]^	2002	Caucasian	Breast cancer	NA	338	345	132/54	90/41			149/39	111/35		
Toruner^[88]^	2001	Caucasian	Bladder cancer	PB	121	121	66/14	22/9			56/11	30/8		

Study groups with more than one race or not explicitly mentioned.

HB = hospital-based group, NA = not available, PB = population-based group.

**Table 3:** Characteristics of the eligible studies for *GSTP1* polymorphisms.

Author	Year	Ethnicity	Cancer type	Source of control	Case (N)	Control (N)	Case (AA/AG + GG)				Control (AA/AG + GG)			
							Smoking	Nonsmoking	Drinking	Non-drinking	Smoking	Nonsmoking	Drinking	Non-drinking
Deek^[24]^	2021	Mixed	Lung cancer	NA	200	200	66/69	29/36			59/40	73/28		
Sciskalska^[94]^	2021	Caucasian	Pancreatic cancer	HB	39	15	11/13	4/11			12/16	9/14		
Xiao^[95]^	2021	Asian	Lung cancer	HB	974	1005	470/210	196/98			311/190	340/162		
Katiyar^[16]^	2020	Caucasian	Head and neck cancer	HB	1250	1250	513/332	227/178	251/188	489/322	193/174	492/391	160/164	525/401
Kudhair^[96]^	2020	Caucasian	Lung cancer	NA	123	129	52/34	28/9			40/11	56/22		
Yamashita^[31]^	2019	Asian	Hypopharyngeal cancer	NA	61	71	22/8	23/9			11/2	44/14		
Ritambhara^[34]^	2019	Caucasian	Lung cancer	HB	120	100	72/40	4/4			10/16	44/30		
Rostami^[30]^	2019	Caucasian	Chronic myeloid leukemia	NA	104	104	30/27	24/23			12/19	26/47		
Thekkepaurakkal^[17]^	2019	Caucasian	Head and neck cancer	HB	389	429	164/156	39/28			143/168	51/66		
Rodrigues-Fleming^[36]^	2018	Caucasian	Colorectal cancer	NA	232	738	57/45	50/80	48/52	59/73	91/115	136/177	111/145	116/147
Negovan	2017	Caucasian	Gastric cancer	NA	101	169	3/8	58/32	14/13	47/27	7/5	78/79	18/22	67/62
Abo-Hashem^[97]^	2016	Caucasian	Liver cancer	NA	40	40	12/8	11/9			4/6	27/3		
Ghosh^[98]^	2016	Caucasian	Gastric cancer	NA	70	82	20/31	9/10	19/15	10/26	5/23	16/38	7/7	14/43
Silva^[42]^	2014	Mixed	Upper aerodigestive tract cancer	PB	116	224	59/39	50/24			16/39	49/52		
Gu^[99]^	2014	Asian	Lung cancer	PB	266	307	63/111	28/56			66/74	66/100		
Yamada^[45]^	2014	Asian	Pancreatic cancer	PB	360	400	49/47	74/71			30/28	99/103		
Jiang^[44]^	2014	Asian	Lung cancer	NA	322	456	19/131	28/144			42/177	51/186		
Matic^[47]^	2013	Caucasian	Bladder cancer	HB	201	122	66/83	18/34			28/38	21/35		
Pandith^[100]^	2013	Caucasian	Bladder cancer	HB	180	210	39/105	10/26			21/135	14/40		
Lv^[101]^	2013	Asian	Lung cancer	HB	116	216	38/37	24/17			53/49	59/55		
Berber^[49]^	2013	Caucasian	Prostate cancer	NA	115	115	32/33	26/24			25/29	37/24		
García-González^[52]^	2012	Caucasian	Gastric cancer	HB	557	557	72/50	126/119			39/36	160/138		
Ramzy^[59]^	2011	Caucasian	Lung cancer	NA	48	42	7/24	2/15			7/7	21/2		
Moaven^[102]^	2010	Caucasian	Esophageal cancer	HB	148	137	33/14	51/50			10/16	65/46		
Li^[66]^	2010	Mixed	Oesophageal cancer	HB	245	288	71/106	21/42	63/102	29/46	77/108	30/65	74/93	33/80
Palma^[67]^	2010	Caucasian	Cervical cancer	PB	81	111	13/15	15/19			17/15	20/25		
Altayli^[70]^	2009	Caucasian	Bladder cancer	PB	135	128	59/35	11/24			29/34	20/27		
Peters^[79]^	2006	Caucasian	Head and neck cancer	NA	692	753	304/246	83/57			254/209	161/124		
Tamer^[5]^	2005	Caucasian	Gastric cancer	HB	70	204	18/16	20/16			41/43	49/71		
Miller^[103]^	2003	Caucasian	Lung cancer	NA	1042	1161	91/109	39/61			92/108	42/58		
Toruner^[88]^	2001	Caucasian	Bladder cancer	PB	121	121	45/35	18/13			45/22	27/11		

Study groups with more than one race or not explicitly mentioned.

HB = hospital-based group, NA = not available, PB = population-based group.

#### 3.1.1. GSTM1 gene polymorphism studies among smokers and drinkers.

Based on the 70 reports on smoking, there were 24 articles for lung cancer, 8 articles for bladder cancer, 6 articles for liver cancer, 5 articles for gastric cancer, 5 articles for cervical cancer, 5 articles for head and neck cancer, 2 articles for esophageal cancer, 2 articles for nasopharyngeal cancer, 2 articles for breast cancer, and 2 articles for prostate cancer; the other types of cancer were not conducted subgroup analysis due to no more than 1 articles involved. Among subgroup of ethnicities, 34 studies were from Caucasian, 30 studies were from Asian, and 6 studies were mixed.

Of the 12 reports on drinking, 3 articles were for gastric cancer, 2 articles were for head and neck cancer, 2 articles were for liver cancer, and 5 other cancers (breast cancer, thyroid cancer, esophageal cancer, colorectal cancer, and lung cancer). Among subgroup of ethnicities, 5 studies were from Caucasian, 5 studies were from Asian, and 2 studies were mixed (Table 4).

**Table 4:** Integral analysis of the association between *GSTM1* polymorphism and cancer risk among smoking and drinking populations.

Comparative model	No.	Z	*P*	OR (95% CI)	Heterogeneity			*Z*	Begg’s test	*t*	Egger’s test	FPRP *P* value	FPRP statistical power	FPRP prior probability					BFDP prior probability		
					Chi-squared	*P*	*I* ^2^							0.25	0.10	0.01	####	0.0001	0.01	####	0.00001
Smoker																					
Overall	70	4.93	**.000**	1.347 (1.196–1.516)	216.91	.000	68.20%	1.06	0.287	−0.16	0.875	.000001	0.984	**0.000**	**0.000**	**0.000**	**0.001**	**0.008**	**0.005**	**0.051**	0.844
Ethnicity																					
Caucasian	34	3.17	**.002**	1.312 (1.109–1.552)	111.13	.000	70.30%	1.57	0.116	−0.20	0.844	.001533	0.972	**0.005**	**0.014**	**0.139**	0.620	0.942	0.829	0.980	1.000
Asian	30	4.78	**.000**	1.515 (1.278–1.797)	63.52	.000	54.30%	0.64	0.521	−0.89	0.382	.000002	0.455	**0.000**	**0.000**	**0.000**	**0.004**	**0.039**	**0.010**	**0.089**	0.907
Mixed	6	0.08	.937	0.984 (0.650–1.488)	25.47	.000	80.40%	0.00	1.000	−0.34	0.750	.939067	0.967	0.744	0.897	0.990	0.999	1.000	0.996	1.000	1.000
Source of control																					
HB	28	2.11	**.035**	1.256 (1.016–1.552)	96.32	.000	72.00%	1.24	0.213	−0.51	0.616	.034759	0.950	**0.099**	0.248	0.784	0.973	0.997	0.984	0.998	1.000
PB	16	1.68	.093	1.261 (0.962–1.652)	62.21	.000	75.90%	0.50	0.620	−0.92	0.371	.092385	0.961	0.236	0.481	0.911	0.990	0.999	0.991	0.999	1.000
Cancer types																					
Lung	24	4.83	**.000**	1.542 (1.294–1.839)	48.90	.001	53.00%	0.92	0.359	0.55	0.591	.000001	0.379	**0.000**	**0.000**	**0.000**	**0.004**	**0.037**	0.008	0.071	0.884
Bladder	8	1.30	.195	1.264 (0.887–1.801)	19.66	.006	64.40%	0.62	0.536	−1.38	0.216	.194656	0.828	0.413	0.679	0.959	0.996	1.000	0.993	0.999	1.000
Liver	6	1.55	.121	1.436 (0.909–2.268)	25.63	.000	80.50%	0.00	1.000	−0.42	0.696	.1207	0.574	0.387	0.654	0.954	0.995	1.000	0.989	0.999	1.000
Head and neck	5	1.63	.104	1.454 (0.926–2.282)	36.31	.000	89.00%	0.24	0.806	−0.52	0.638	.103586	0.554	0.359	0.627	0.949	0.995	0.999	0.988	0.999	1.000
Cervical	5	0.67	.502	1.102 (0.830–1.463)	5.17	.27	22.70%	0.73	0.462	1.53	0.223	.501669	0.984	0.605	0.821	0.981	0.998	1.000	0.997	1.000	1.000
Stomach	5	1.91	.057	1.656 (0.986–2.782)	8.67	.070	53.90%	0.73	0.462	1.48	0.236	.56683	0.354	0.324	0.590	0.941	0.994	0.999	0.981	0.998	1.000
Nasopharyngeal	2	3.53	**.000**	3.631 (1.775–7.428)	0.21	.65	0.00%					.000414	0.008	**0.138**	0.325	0.841	0.982	0.998	**0.695**	0.958	1.000
Breast	2	0.23	.819	1.048 (0.702–1.564)	0.54	.463	0.00%					.818464	0.960	0.719	0.885	0.988	0.999	1.000	0.996	1.000	1.000
Prostate	2	0.18	.860	0.960 (0.608–1.515)	0.22	.64	0.00%					.860788	0.941	0.733	0.892	0.989	0.999	1.000	0.996	1.000	1.000
Esophageal	2	0.88	.377	0.822 (0.532–1.270)	0.73	.394	0.00%					.377167	0.827	0.578	0.804	0.978	0.998	1.000	0.995	0.999	1.000
No smoker																					
Overall	70	6.09	**.000**	1.423 (1.270–1.594)	152.42	.000	54.70%	1.58	0.114	1.83	0.071	.0000000	0.819	**0.000**	**0.000**	**0.000**	**0.000**	**0.000**	**0.000**	**0.000**	**0.010**
Ethnicity																					
Caucasian	34	4.34	**.000**	1.464 (1.232–1.739)	83.74	.000	60.60%	1.19	0.236	1.73	0.092	.000014	0.609	**0.000**	**0.000**	**0.002**	**0.023**	**0.190**	**0.061**	**0.396**	0.985
Asian	30	5.79	**.000**	1.386 (1.241–1.548)	56.48	.002	48.70%	1.39	0.164	1.13	0.267	.0000000	0.919	**0.000**	**0.000**	**0.000**	**0.000**	**0.000**	**0.000**	**0.001**	**0.059**
Mixed	6	0.95	.343	1.226 (0.804–1.870)	11.43	.044	56.20%	0.75	0.452	−0.34	0.752	.344173	0.825	0.556	0.790	0.976	0.998	1.000	0.995	0.999	1.000
Source of control																					
HB	28	3.47	**.001**	1.432 (1.169–1.754)	65.63	.000	58.90%	0.69	0.489	1.23	0.230	.000521	0.673	**0.002**	**0.007**	**0.071**	0.436	0.886	**0.618**	0.942	0.999
PB	16	2.78	**.005**	1.442 (1.114–1.867)	45.19	.000	66.80%	0.23	0.822	−0.12	0.908	.005479	0.618	**0.026**	**0.074**	0.468	0.899	0.989	0.918	0.991	1.000
Cancer types																					
Lung	24	7.85	**.000**	1.699 (1.488–1.939)	30.60	.133	24.80%	1.91	0.056	2.06	0.052	.0000000	0.032	**0.000**	**0.000**	**0.000**	**0.000**	**0.000**	**0.000**	**0.000**	**0.000**
Bladder	8	2.40	**.017**	1.329 (1.053–1.676)	4.76	.689	0.00%	0.62	0.536	−0.22	0.834	.016257	0.847	**0.054**	**0.147**	0.655	0.950	0.995	0.968	0.997	1.000
Liver	6	1.07	.284	1.515 (0.709–3.239)	44.89	.000	88.90%	0.38	0.707	1.51	0.206	.283923	0.490	0.635	0.839	0.983	0.998	1.000	0.992	0.999	1.000
Head and neck	5	1.50	.134	1.150 (0.958–1.380)	4.14	.387	3.40%	0.24	0.806	−0.59	0.594	.132975	0.998	0.286	0.545	0.930	0.993	0.999	0.995	1.000	1.000
Stomach	5	2.26	**.024**	1.568 (1.062–2.314)	8.44	.077	52.60%	1.71	0.086	6.86	0.006	.023493	0.412	**0.146**	0.339	0.850	0.983	0.998	0.968	0.997	1.000
Cervical	5	2.08	**.037**	1.617 (1.029 –2.542)	10.58	.032	62.20%	0.24	0.806	0.09	0.937	.037328	0.372	0.231	0.474	0.908	0.990	0.999	0.976	0.998	1.000
Nasopharyngeal	2	3.25	**.001**	2.554 (1.452–4.493)	0.53	.467	0.00%					.00114	0.032	**0.095**	0.240	0.777	0.972	0.997	**0.767**	0.971	1.000
Breast	2	1.94	.053	0.705 (0.495–1.004)	0.35	.552	0.00%					.052639	0.622	0.203	0.432	0.893	0.988	0.999	0.983	0.998	1.000
Prostate	2	0.46	.649	1.154 (0.623–2.138)	0.28	.596	0.00%					.648912	0.798	0.709	0.880	0.998	0.999	1.000	0.995	0.999	1.000
Esophageal	2	0.08	.934	0.945 (0.247–3.607)	3.18	.075	68.50%					.934027	0.695	0.801	0.924	0.993	0.999	1.000	0.992	0.999	1.000
Drinker																					
Overall	12	2.33	**.020**	1.748 (1.093–2.797)	108.21	.000	89.80%	0.48	0.631	0.20	0.849	.019882	0.262	**0.186**	0.406	0.883	0.987	0.999	0.962	0.996	1.000
Ethnicity																					
Caucasian	5	3.48	**.001**	2.873 (1.584–5.209)	33.24	.000	88.00%	0.73	0.462	0.19	0.863	.000508	0.016	**0.086**	0.221	0.757	0.969	0.997	0.660	0.952	0.999
Asian	5	1.18	.237	1.518 (0.760–3.033)	13.70	.008	70.85%	0.24	0.806	0.44	0.693	.237229	0.487	0.594	0.814	0.980	0.998	1.000	0.991	0.999	1.000
Mixed	2	0.82	.411	0.695 (0.292–1.654)	7.77	.005	87.10%					.410797	0.537	0.696	0.873	0.987	0.999	1.000	0.992	0.999	1.000
Source of control																					
HB	5	1.47	.142	1.853 (0.813–4.224)	35.42	.000	88.70%	0.24	0.806	−0.39	0.721	.142321	0.308	0.581	0.806	0.979	0.998	1.000	0.988	0.999	1.000
PB	5	1.72	.086	2.099 (0.900–4.892)	65.31	.000	93.90%	1.22	0.221	1.11	0.350	.085884	0.218	0.541	0.780	0.975	0.997	1.000	0.985	0.998	1.000
Cancer types																					
Stomach	3	2.68	**.007**	1.817 (1.175–2.809)	3.28	.194	39.10%	1.04	0.296	6.78	0.930	.007214	0.194	**0.100**	0.251	0.786	0.974	0.997	0.922	0.992	1.000
Head and neck	2	4.38	**.000**	3.747 (2.076–6.764)	4.26	.039	76.50%					.000012	0.001	**0.029**	**0.081**	0.493	0.907	0.990	**0.108**	**0.550**	0.992
Liver	2	2.44	**.015**	4.244 (1.327–13.574)	6.45	.011	84.50%					.014816	0.040	0.528	0.770	0.974	0.997	1.000	0.971	0.997	1.000
No drinker																					
Overall	12	1.67	.096	1.215 (0.966–1.527)	29.31	.002	62.50%	0.62	0.537	−0.82	0.431	.094918	0.965	0.228	0.470	0.907	0.990	0.999	0.992	0.999	1.000
Ethnicity																					
Caucasian	5	1.29	.198	1.220 (0.901–1.650)	10.70	.030	62.65%	0.73	0.462	−0.63	0.575	.196746	0.910	0.393	0.661	0.955	0.995	1.000	0.994	0.999	1.000
Asian	5	1.57	.118	1.368 (0.924–2.025)	8.43	.077	52.60%	−0.24	1.000	0.22	0.842	.117379	0.667	0.342	0.609	0.945	0.994	0.999	0.990	0.999	1.000
Mixed	2	0.32	.751	0.813 (0.225–2.932)	9.25	.002	89.20%					.751747	0.619	0.785	0.916	0.992	0.999	1.000	0.992	0.999	1.000
Source of control																					
HB	5	1.05	.294	1.276 (0.809–2.011)	12.09	.017	66.90%	0.24	0.806	−0.36	0.743	.293653	0.757	0.538	0.777	0.975	0.997	1.000	0.994	0.999	1.000
PB	5	1.17	.234	1.231 (0.869–1.745)	10.59	.032	62.20%	0.24	0.806	−0.08	0.940	.243046	0.867	0.457	0.716	0.965	0.996	1.000	0.994	0.999	1.000
Cancer types																					
Stomach	3	1.75	.080	1.446 (0.957–2.185)	2.13	.345	5.90%	0.00	1.000	−0.85	0.550	.079941	0.569	0.296	0.558	0.933	0.993	0.999	0.987	0.999	1.000
Head and neck	2	0.14	.888	1.047 (0.552–1.986)	8.20	.004	87.80%					.888174	0.864	0.755	0.902	0.990	0.999	1.000	0.995	0.999	1.000
Liver	2	2.36	**.018**	1.748 (1.100–2.779)	0.01	.903	0.00%					.018226	0.259	0.174	0.388	0.875	0.986	0.999	0.960	0.996	1.000

Results shown in bold indicate the *P* value < .05, the statistically significant noteworthiness at 0.2 level by FPRP or 0.8 level by BFDP calculations.

BFDP = Bayesian False Discovery Probability, CI = confidence interval, FPRP = false positive report probability, HB = hospital-based, OR = odds ratio, PB = population-based.

#### 3.1.2. GSTT1 gene polymorphism studies among smokers and drinkers.

Of the 49 articles on smoking, 13 articles were for lung cancer, 6 articles were for bladder cancer, 5 articles were for gastric cancer, 5 articles were for cervical cancer, 4 articles were for liver cancer, 2 articles were for head and neck cancer, 2 articles were for breast cancer, 2 articles were for pancreatic cancer, 2 articles were for prostate cancer, as well as other 8 articles that were not performed subgroup analysis for the same reasons as above. Among subgroup of ethnicities, 30 studies were from Caucasian, 14 studies were from Asian, and 5 studies were mixed.

Of the 8 studies on drinking, 2 articles were for gastric cancer and 6 articles were for other cancers (breast cancer, thyroid cancer, esophageal cancer, colorectal cancer, lung cancer, and liver cancer). Among subgroup of ethnicities, 4 studies were from Caucasian, 2 studies were from Asian, and 2 studies were mixed (Table 5).

**Table 5:** Integral analysis of the association between *GSTT1* polymorphism and cancer risk among smoking ang drinking populations.

Comparative model	No.	*Z*	*P*	OR (95% CI)	Heterogeneity			Z	Begg’s Test	*t*	Egger’s test	FPRP *P* value	FPRP statistical power	FPRP prior probability					BFDP prior probability		
					Chi-squared	*P*	*I* ^2^							0.25	0.10	0.01	####	0.0001	0.01	0.001	0.000001
Smoker																					
Overall	49	3.04	**.002**	1.356 (1.114–1.651)	237.36	0.000	79.80%	2.30	0.021	2.16	0.036	.0024	0.843	**0.009**	**0.025**	0.222	0.742	0.966	0.867	0.985	1.000
Ethnicity																					
Caucasian	30	1.97	**.049**	1.318 (1.001–1.736)	161.36	0.000	82.00%	1.43	0.153	1.22	0.232	.0495	0.821	**0.153**	0.351	0.856	0.984	0.998	0.985	0.999	1.000
Asian	14	2.78	**.005**	1.701 (1.170–2.473)	53.85	0.000	75.90%	1.09	0.274	1.47	0.166	.0054	0.255	**0.060**	**0.160**	0.677	0.955	0.995	0.905	0.990	1.000
Mixed	5	0.08	.936	0.985 (0.683–1.421)	12.64	0.013	68.40%	0.73	0.462	.55	0.620	.9356	0.982	0.741	0.896	0.990	0.999	1.000	0.997	1.000	1.000
Source of control																					
HB	17	3.53	**.000**	1.699 (1.266–2.280)	52.09	0.000	69.30%	1.77	0.077	1.61	0.128	.0004	0.203	**0.006**	**0.018**	**0.167**	0.670	0.953	**0.532**	0.920	0.999
PB	14	1.14	.256	1.329 (0.814–2.171)	114.42	0.000	88.60%	0.44	0.661	.42	0.679	.256	0.686	0.528	0.771	0.974	0.997	1.000	0.993	0.999	1.000
Cancer types																					
Lung	13	1.79	.073	1.573 (0.959–2.581)	101.50	0.000	88.20%	1.89	0.059	1.53	0.154	.073	0.425	0.340	0.607	0.944	0.994	0.999	0.984	0.998	1.000
Bladder	6	0.01	.991	1.003 (0.646–1.555)	12.59	0.028	60.30%	0.38	0.707	.70	0.525	.9893	0.964	0.755	0.902	0.990	0.999	1.000	0.996	1.000	1.000
Stomach	5	2.00	**.045**	1.944 (1.015–3.724)	11.09	0.026	63.90%	1.22	0.221	2.44	0.092	.045	0.217	0.384	0.651	0.954	0.995	1.000	0.978	0.998	1.000
Cervical	5	0.44	.658	1.077 (0.775–1.498)	5.32	0.256	24.80%	−0.24	1.000	2.48	0.089	.6595	0.975	0.670	0.859	0.985	0.999	1.000	0.997	1.000	1.000
Liver	4	0.68	.498	1.300 (0.608–2.776)	24.92	0.000	88.00%	1.02	0.308	−2.74	0.111	.4979	0.644	0.699	0.874	0.987	0.999	1.000	0.993	0.999	1.000
Head and neck	2	0.09	.928	1.033 (0.506–2.111)	11.37	0.001	91.20%					.9291	0.847	0.767	0.908	0.991	0.999	1.000	0.995	0.999	1.000
Breast	2	1.78	.074	1.524 (0.959–2.420)	0.24	0.624	0.00%					.0741	0.473	0.320	0.585	0.939	0.994	0.999	0.985	0.998	1.000
Pancreas	2	3.31	**.001**	3.749 (0.254–55.420)	23.75	0.000	95.80%					.3362	0.253	0.800	0.923	0.992	0.999	1.000	0.990	0.999	1.000
Prostate	2	3.32	**.001**	2.847 (1.536–5.276)	0.07	0.789	0.00%					.0009	0.021	**0.113**	0.277	0.808	0.977	0.998	**0.752**	0.968	1.000
No smoker																					
Overall	49	2.19	**.028**	1.103 (1.011–1.204)	72.78	0.012	34.00%	0.78	0.433	.98	0.331	.0283	1.000	**0.078**	0.203	0.737	0.966	0.996	0.991	0.999	1.000
Ethnicity																					
Caucasian	30	1.19	.234	1.077 (0.953–1.217)	40.38	0.078	28.20%	1.61	0.108	1.30	0.206	.2342	1.000	0.413	0.678	0.959	0.996	1.000	0.989	0.999	1.000
Asian	14	1.98	**.047**	1.165 (1.002–1.355)	15.75	0.263	17.50%	0.33	0.743	−.58	0.575	.0476	0.999	**0.125**	0.300	0.825	0.979	0.998	0.997	1.000	1.000
Mixed	5	0.64	.523	1.190 (0.698–2.029)	15.97	0.003	744.90%	−0.24	1.000	.460	0.677	.5228	0.802	0.662	0.854	0.985	0.998	1.000	0.995	0.999	1.000
Source of control																					
HB	17	1.47	.142	1.121 (0.953–1.306)	16.10	0.446	0.60%	0.29	0.773	.60	0.556	.1427	1.000	0.300	0.562	0.934	0.993	0.999	0.996	1.000	1.000
PB	14	3.56	**.000**	1.348 (1.144–1.589)	22.39	0.050	42.00%	0.11	0.913	−.10	0.920	.0004	0.899	**0.001**	**0.004**	**0.039**	0.293	0.806	**0.578**	0.932	0.999
Cancer types																					
Lung	13	2.32	**.020**	1.233 (1.033–1.472)	13.84	0.311	13.30%	0.18	0.855	−.27	0.794	.0205	0.985	**0.059**	**0.158**	0.673	0.954	0.995	0.978	0.998	1.000
Bladder	6	1.25	.210	1.216 (0.896–1.652)	4.41	0.492	0.00%	1.13	0.260	1.47	0.215	.211	0.910	0.410	0.676	0.958	0.996	1.000	0.994	0.999	1.000
Stomach	5	0.88	.380	1.134 (0.856–1.502)	6.17	0.187	35.20%	0.73	0.462	−.73	0.946	.3805	0.974	0.539	0.778	0.975	0.997	1.000	0.996	1.000	1.000
Cervical	5	1.69	.092	0.765 (0.561–1.045)	7.71	0.127	44.20%	−0.24	1.000	−.01	0.996	.0923	0.806	0.256	0.507	0.919	0.991	0.999	0.990	0.999	1.000
Liver	4	0.91	.364	1.297 (0.740–2.276)	6.48	0.090	53.70%	−0.34	1.000	−.12	0.916	.3647	0.694	0.612	0.826	0.981	0.998	1.000	0.994	0.999	1.000
Head and neck	2	0.14	.890	0.982 (0.757–1.274)	0.20	0.659	0.00%					.8912	0.998	0.728	0.889	0.989	0.999	1.000	0.998	1.000	1.000
Breast	2	1.16	.245	1.240 (0.863–1.781)	0.60	0.437	0.00%					.2442	0.849	0.463	0.721	0.966	0.997	1.000	0.994	0.999	1.000
Pancreas	2	0.83	.407	1.170 (0.807–1.697)	0.50	0.480	0.00%					.4079	0.905	0.575	0.802	0.978	0.998	1.000	0.996	1.000	1.000
Prostate	2	0.50	.618	1.215 (0.566–2.613)	0.04	0.846	0.00%					.6182	0.705	0.725	0.888	0.989	0.999	1.000	0.994	0.999	1.000
Drinker																					
Overall	8	2.22	**.026**	1.423 (1.042–1.942)	20.46	0.005	65.80%	0.12	0.902	.65	0.538	.0262	0.630	**0.111**	0.272	0.804	0.976	0.998	0.974	0.997	1.000
Ethnicity																					
Caucasian	4	2.06	**.039**	1.543 (1.021–2.332)	8.98	0.030	66.60%	0.34	0.734	−.86	0.481	.0396	0.447	0.210	0.444	0.898	0.989	0.999	0.978	0.998	1.000
Asian	2	1.99	**.047**	2.250 (1.011–5.008)	0.17	0.684	0.00%					.047	0.160	0.468	0.725	0.967	0.997	1.000	0.979	0.998	1.000
Mixed	2	0.11	.911	1.015 (0.787–1.307)	1.96	0.161	49.10%					.9081	0.999	0.732	0.891	0.989	0.999	1.000	0.998	1.000	1.000
Source of control																					
HB	3	3.68	**.000**	1.615 (1.252–2.084)	1.85	0.396	0.00%	0.00	1.000	.45	0.728	.0002	0.285	**0.002**	**0.007**	**0.074**	0.445	0.889	**0.411**	0.876	0.999
PB	3	1.19	.236	1.469 (0.778–2.771)	12.41	0.002	83.90%	0.00	1.000	.99	0.503	.2349	0.526	0.573	0.801	0.978	0.998	1.000	0.991	0.999	1.000
Cancer type																					
Stomach	2	2.30	**.021**	1.877 (1.098–3.209)	0.49	0.486	0.00%	1.04	0.296	1.05	0.486	.0214	0.206	0.237	0.483	0.911	0.990	0.999	0.964	0.996	1.000
No drinker																					
Overall	8	2.03	**.042**	1.458 (1.014–2.098)	26.23	0.000	73.30%	1.36	0.174	1.17	0.286	.0423	0.561	**0.184**	0.404	0.882	0.987	0.999	0.980	0.998	1.000
Ethnicity																					
Caucasian	4	1.46	.143	1.687 (0.838–3.397)	22.49	0.000	86.70%	0.34	0.734	.93	0.451	.1431	0.371	0.536	0.776	0.974	0.997	1.000	0.988	0.999	1.000
Asian	2	0.04	.956	1.010 (0.645–1.583)	0.15	0.696	0.00%					.9654	0.958	0.751	0.901	0.990	0.999	1.000	0.996	1.000	1.000
Mixed	2	2.31	.021	1.574 (1.070–2.314)	1.39	0.238	28.20%					.021	0.403	**0.135**	0.320	0.838	0.981	0.998	0.965	0.996	1.000
Source of control																					
HB	3	2.13	**.033**	1.193 (0.964–1.476)	3.08	0.215	35.00%	0.00	1.000	.71	0.607	.1042	0.983	0.241	0.488	0.913	0.991	0.999	0.993	0.999	1.000
PB	3	2.19	**.029**	2.460 (1.099–5.507)	8.80	0.012	77.30%	0.00	1.000	1.19	0.446	.0286	0.114	0.428	0.692	0.961	0.996	1.000	0.972	0.997	1.000
Cancer type																					
Stomach	2	1.37	.172	1.804 (0.773–4.207)	2.02	0.156	50.40%					.172	0.335	0.607	0.822	0.981	0.998	1.000	0.989	0.999	1.000

Results shown in bold indicate the *P* value < .05, the statistically significant noteworthiness at 0.2 level by FPRP or 0.8 level by BFDP calculations.

BFDP = Bayesian False Discovery Probability, CI = confidence interval, FPRP = false positive report probability, HB = hospital-based, OR = odds ratio, PB = population-based.

#### 3.1.3. GSTP1 gene polymorphism studies among smokers and drinkers.

Of the 31 reports on smoking, 9 articles were for lung cancer, 4 articles were for bladder cancer, 4 articles were for gastric cancer, 3 articles were for head and neck cancer, 2 articles were for pancreatic cancer, and 9 articles without subgroup analysis for the same reasons as above. Among subgroup of ethnicities, 22 studies were from Caucasian, 6 studies were from Asian, and 3 studies were mixed.

Of the 5 studies on drinking, articles were for gastric cancer, head and neck cancer, esophageal cancer, and colorectal cancer; 4 studies were from Caucasian and 1 study was from Asian (Table 6).

**Table 6:** Integral analysis of the association between *GSTP1rs1695* polymorphism and cancer risk among smoking and drinking populations.

Comparative model	No.	*Z*	*P*	OR (95% CI)	Heterogeneity			*Z*	Begg’s Test	*t*	Egger’s test	FPRP *P* value	FPRP statistical power	FPRP prior probability					BFDP prior probability		
					Chi-squared	*P*	*I* ^2^							0.25	0.10	0.01	####	0.0001	0.01	####	0.000001
Smoker																					
Overall	31	1.65	.098	0.870 (0.737–1.026)	75.50	0.000	60.30%	0.17	0.865	0.32	0.751	.097931	0.999	0.227	0.469	0.907	0.990	0.999	0.994	0.999	0.999994
Ethnicity																					
Caucasian	22	2.29	**.022**	0.800 (0.661–0.968)	43.84	0.002	52.10%	0.17	0.866	−0.09	0.928	.021767	0.970	0.063	0.168	0.690	0.957	0.996	0.978	0.998	0.999978
Asian	6	0.72	.474	1.142 (0.794–1.642)	13.36	0.020	62.60%	0.00	1.000	2.04	0.111	.473571	0.929	0.605	0.821	0.981	0.998	1.000	0.996	1.000	0.999996
Mixed	3	0.52	.601	0.793 (0.332–1.892)	15.59	0.000	87.20%	0.00	1.000	−1.11	0.468	.601131	0.652	0.734	0.892	0.989	0.999	1.000	0.993	0.999	0.999993
Source of control																					
HB	12	4.64	**.000**	0.747 (0.661–0.845)	15.80	0.149	30.40%	0.62	0.537	−0.80	0.443	.000004	0.965	0.000	0.000	0.000	0.004	0.035	0.021	0.179	0.956293
PB	6	0.43	.668	0.881 (0.493–1.574)	23.07	0.000	78.30%	0.38	0.707	−0.75	0.496	.668712	0.827	0.708	0.879	0.988	0.999	1.000	0.995	0.999	0.999995
Cancer types																					
Lung	9	1.03	.303	1.195 (0.851–1.676)	28.82	0.000	72.20%	0.94	0.348	1.71	0.130	.301964	0.906	0.500	0.750	0.971	0.997	1.000	0.995	1.000	0.999995
Stomach	4	1.32	.187	0.758 (0.503–1.143)	5.27	0.153	43.00%	0.34	0.734	0.52	0.653	.186105	0.730	0.433	0.696	0.962	0.996	1.000	0.992	0.999	0.999992
Bladder	4	1.01	.310	0.741 (0.415–1.323)	10.41	0.015	71.20%	0.34	0.734	0.76	0.525	.310791	0.640	0.593	0.810	0.980	0.998	1.000	0.993	0.999	0.999993
Head and neck	3	2.35	**.019**	0.833 (0.715–0.970)	3.14	0.208	36.30%	0.00	1.000	−0.09	0.943	.018668	0.998	0.053	0.144	0.649	0.949	0.995	0.980	0.998	0.999979
Pancreas	2	0.04	.969	0.989 (0.564–1.732)	0.05	0.820	0.00%					.969138	0.916	0.760	0.905	0.991	0.999	1.000	0.995	1.000	0.999995
No smoker																					
Overall	31	0.57	.569	1.051 (0.886–1.247)	69.34	0.000	56.70%	1.39	0.163	1.28	0.210	.568557	1.000	0.630	0.837	0.983	0.998	1.000	0.998	1.000	0.999998
Ethnicity																					
Caucasian	22	0.30	.766	1.033 (0.834–1.279)	47.30	0.001	55.60%	1.75	0.080	1.35	0.192	.765776	1.000	0.697	0.873	0.987	0.999	1.000	0.998	1.000	0.999998
Asian	6	0.78	.437	1.080 (0.889–1.312)	3.10	0.684	0.00%	0.00	1.000	0.21	0.841	.438233	1.000	0.568	0.798	0.977	0.998	1.000	0.998	1.000	0.999998
Mixed	3	0.17	.867	1.103 (0.351–3.464)	18.47	0.000	89.20%	0.00	1.000	0.52	0.695	.866660	0.701	0.788	0.918	0.992	0.999	1.000	0.993	0.999	0.999993
Source of control																					
HB	12	0.17	.863	0.988 (0.861–1.133)	9.38	0.587	0.00%	0.21	0.837	−0.37	0.721	.862816	1.000	0.721	0.886	0.988	0.999	1.000	0.999	1.000	0.999999
PB	6	0.23	.819	0.970 (0.751–1.254)	9.60	0.087	47.90%	0.75	0.452	0.72	0.513	.816166	0.998	0.710	0.880	0.988	0.999	1.000	0.998	1.000	0.999998
Cancer types																					
Lung	9	1.75	.080	1.444 (0.957–2.180)	27.95	0.000	71.40%	0.94	0.348	1.61	0.151	.080413	0.572	0.297	0.559	0.933	0.993	0.999	0.987	0.999	0.999987
Stomach	4	1.54	.124	0.692 (0.433–1.106)	7.18	0.066	58.20%	−0.34	1.000	−2.26	0.152	.123833	0.562	0.398	0.665	0.956	0.995	1.000	0.989	0.999	0.999989
Bladder	4	1.10	.271	1.288 (0.821–2.020)	1.24	0.743	0.00%	0.34	0.734	0.60	0.607	.270317	0.747	0.521	0.765	0.973	0.997	1.000	0.994	0.999	0.999994
Head and neck	3	0.97	.334	0.909 (0.748–1.104)	3.01	0.223	33.50%	1.04	0.296	−2.52	0.241	.335948	0.999	0.502	0.752	0.971	0.997	1.000	0.997	1.000	0.999997
Pancreas	2	0.12	.904	0.975 (0.649–1.466)	0.74	0.389	0.00%					.903162	0.966	0.737	0.894	0.989	0.999	1.000	0.996	1.000	0.999996
Drinker																					
Overall	5	1.52	.130	0.853 (0.695–1.048)	4.59	0.332	12.90%	0.24	0.806	0.27	0.802	.130112	0.991	0.283	0.542	0.929	0.992	0.999	0.994	0.999	0.999994
Ethnicity																					
Caucasian	4	2.33	**.020**	0.758 (0.600–0.957)	0.21	0.976	24.50%	0.34	0.734	−0.47	0.687	.019831	0.860	0.065	0.172	0.695	0.958	0.996	0.973	0.997	0.999972
Source of control																					
HB	2	1.16	.248	0.868 (0.683–1.104)	4.49	0.034	77.70%					.248631	0.984	0.431	0.695	0.962	0.996	1.000	0.996	1.000	0.999996
NA	3	1.02	.308	0.813 (0.547–1.210)	0.03	0.986	0.00%	1.04	0.296	−1.18	0.447	.307526	0.836	0.525	0.768	0.973	0.997	1.000	0.995	0.999	0.999995
No drinker																					
Overall	5	2.14	**.032**	0.840 (0.717–0.985)	2.22	0.696	0.00%	0.24	0.806	0.27	0.802	.031817	0.998	0.087	0.223	0.760	0.970	0.997	0.986	0.999	0.999986
Ethnicity																					
Caucasian	4	1.86	.063	0.855 (0.726– 1.008)	1.54	0.674	0.00%	0.34	0.734	−0.47	0.687	.062163	0.998	0.157	0.359	0.860	0.984	0.998	0.992	0.999	0.999992
Source of control																					
HB	2	1.85	.065	0.842 (0.701–1.011)	0.70	0.403	0.00%					.065362	0.994	0.165	0.372	0.867	0.985	0.998	0.991	0.999	0.999991
NA	3	1.09	.276	0.837 (0.608–1.153)	1.51	0.469	0.00%	0.00	1.000	−0.47	0.721	.276236	0.918	0.474	0.730	0.968	0.997	1.000	0.995	0.999	0.999995

Results shown in bold indicate the *P* value < .05, the statistically significant noteworthiness at 0.2 level by FPRP or 0.8 level by BFDP calculations.

BFDP = Bayesian False Discovery Probability, CI = confidence interval, FPRP = false positive report probability, HB = hospital-based, OR = odds ratio, PB = population-based.

### 3.2. Quantitative synthesis

#### 3.2.1. The relationship between GSTM1 polymorphism and cancer risks alone and in combination with smoking or drinking.

*GSTM1* (null/present) might rise the overall cancer risks in both smokers (*I*^2^ = 68.20%, OR = 1.347, 95% CI: 1.196–1.516, *P < *.001) and nonsmokers (*I^2^* = 54.70%, OR = 1.423, 95% CI: 1.270–1.594, *P < *.001). And subgroup results demonstrated that *GSTM1*-null might increase cancer risks regardless of ethnicities (Caucasian and Asian) or whether smoking. Subgroup results also showed that high risk of lung and nasopharyngeal cancer could be found in both smokers and nonsmokers, but *GSTM1*-null could lead to increased cancer risks of the stomach, the cervical and the bladder even nonsmoking (Table 4).

The overall finding showed significant statistical association was found between cancer risks and *GSTM1*-null in drinkers (*I*^2^ = 89.8%, OR = 2.33, 95% CI: 1.093–2.797, *P = *.02). Subgroup results demonstrated that alcohol consumption might increase the cancer risks of *GSTM1*-null carriers among Caucasians with a 2.873-fold, but no association was found among Asians. Subgroup results also showed that drinking increased cancer risks of the stomach with a 1.817-fold, the head and neck with a 3.747-fold and the liver with a 4.244-fold among *GSTM1*-null carriers (Table 4).

#### 3.2.2. The relationship between GSTT1 polymorphism and cancer risks alone and in combination with smoking or drinking.

Significant positive correlation was found between *GSTT1* (null/present) and the overall cancer risks among smokers (*I*^2^ = 79.80%, OR = 1.356, 95% CI: 1.114–1.651, *P = *.002) and nonsmokers (*I*^2^ = 34.00%, OR = 1.103, 95% CI: 1.011–1.204, *P = *.028). Subgroup analysis found that smoking might increase the cancer risks in Caucasian smokers with a 1.318-fold and in Asian smokers with a 1.701-fold. The hospital-based source of control group was found with a 1.699-fold cancer risks among smokers, whereas, in contrast, the population-based nonsmokers were found with a 1.348-fold cancer risks. Subgroup results also showed that smoking was highly associated with cancer risks of the stomach, the pancreas and the prostate with a 1.944-fold, a 3.749-fold, and a 2.847-fold, respectively, compared with nonsmokers (Table 5).

Significant association was also found between *GSTT1* (null/present) and the overall cancer risks among drinkers (*I*^2^ = 65.80%, OR = 1.423, 95% CI: 1.042–1.942, *P = *.026) and nondrinkers (*I*^2^ = 373.30%, OR = 1.458, 95% CI: 1.014–2.098, *P = *.021). Subgroup analysis found that drinking might be more likely to increase the cancer susceptibility among Asian *GSTT1*-null carriers by 2.250 times compared with 1.543 times among Caucasians. *GSTT1*-null was associated with increased cancer risks among nonsmokers in both hospital-based and population-based control group. Subgroup results also showed that alcohol consumption might increase the cancer risks of the stomach to 1.877 times compared with nondrinkers (Table 5).

#### 3.2.3. The relationship between GSTP1 polymorphism and cancer risks alone and in combination with smoking or drinking.

*GSTP1* polymorphism (AG + GG/AA) was not statistically associated with the overall cancer risks in either smokers or nonsmokers. However, subgroup analysis indicated that *GSTP1* (AG + GG) variations might reduce the risk of cancers among Caucasian smokers. And the negative correlation might also exit in the risk of head and neck cancer in combination with smoking (OR = .833, 95% CI: 0.715–0.970, *P = *.019) (Table 6).

A reduced cancer risks was found among nondrinkers with *GSTP1* (AG + GG) variations (*I*^2^ = 0.00%, OR = .840, 95% CI: 0.717–0.985, *P = *.032). Interestingly, subgroup results indicated that *GSTP1* (AG + GG) variations in combination with drinking might have a protective effect among Caucasians (Table 6).

In summary, the main results of this study were presented in Table 7. Positive correlation was found between *GSTM1* (null/present) and the overall cancer risks among smokers (OR = 1.347, 95% CI: 1.196–1.516, *P* < .001) and drinkers (OR = 1.748, 95% CI: 1.093–2.797, *P = *.02). *GSTT1* (null/present) might be strongly associated with human cancer risks increases (for smokers: OR = 1.356, 95% CI: 1.114–1.651, *P = *.002; for nonsmokers: OR = 1.103, 95% CI: 1.011–1.204, *P = *.028; for drinkers: OR = 1.423, 95% CI: 1.042–1.942, *P = *.026; for nondrinkers: OR = 1.458, 95% CI: 1.014–2.098, *P = *.042). It has not been found whether *GSTP1rs1695* (AG + GG/AA) might increase cancer risks of human with the living habits of smoking or drinking, but an interesting result indicated that it might have a protect effect of nondrinkers’ cancer risks.

**Table 7:** Comprehensive analysis of the association between GSTs and cancer risk.

Living habits	*GSTM1*			*GSTT1*			*GSTP1rs1695*		
	*Z*	*P*	OR (95% CI)	*Z*	*P*	OR (95% CI)	*Z*	*P*	OR (95% CI)
Smoking	4.93	**.000**	1.347 (1.196–1.516)	3.04	**.002**	1.356 (1.114–1.651)	1.65	.098	0.870 (0.737–1.026)
No-smoking	6.09	**.000**	1.423 (1.270–1.594)	2.19	**.028**	1.103 (1.011–1.204)	0.57	.569	1.051 (0.886–1.247)
Drinking	2.33	**.020**	1.748 (1.093–2.797)	2.22	**.026**	1.423 (1.042–1.942)	1.52	.130	0.853 (0.695–1.048)
No-drinking	1.67	.096	1.215 (0.966–1.527)	2.03	**.042**	1.458 (1.014–2.098)	2.14	**.032**	0.840 (0.717–0.985)

Results shown in bold indicate the *P* value < 0.05.

### 3.3. Publication bias

The results of publication bias for *GSTM1, GSTT1*, and *GSTP1* gene polymorphisms and cancer risks were shown in Tables 4–6, respectively. Egger test for *GSTT1* combined with smoking showed publication bias of 0.036 (Table 5).

### 3.4. Sensitivity analysis

No statistically significant variables were found in the sensitivity analysis of *GSTM1, GSTT1* and *GSTP1* gene polymorphisms. The meta-regression analysis found that the year, the ethnicity, and the source of control population were not associated with experimental heterogeneity.

### 3.5. FPRP and BFDP test

The FPRP and BFDP analysis values of GSTs gene polymorphisms and smoking or drinking were shown in Tables 4–6, respectively. According to the results of the FPRP analysis, a part of the results in GSTs polymorphisms models were noteworthy in the FPRP test at the OR of 1.5 with prior probabilities of 0.25 and 0.1, but very few results were noteworthy in the BFDP test at the OR of 1.5 with prior probabilities of 0.01, 0.001 and 0.00001, which suggested that the results of this study should be interpreted with caution and euphemism.

## 4. Discussion

Numerous studies have shown that smoking and drinking contributed to the development of cancers, but cancers did not occur in all smokers and drinkers. The occurrence of cancers had a certain probability which might closely relate to the genetic metabolism of genes. Smoking and drinking might have a synergistic effect with the biological metabolic enzymes regulated by GSTs genes. And it was shown in this study that *GSTM1, GSTT1,* and *GSTP1* polymorphisms, alone or in combination with smoking or drinking, might affect the overall cancer risks differently, and the effects might be related to ethnicities.

For *GSTM1*, plenty of meta-analyses have shown that *GSTM1*-null could increase cancer risks in some specific organs among smokers or drinkers^[104–108]^; while some scholars suggested that this cancer susceptibility might not be augmented by smoking or drinking.^[109,110]^ The outcome of this study indicated that *GSTM1*-null, both alone and in combination with smoking or drinking, was associated with the overall increased cancer risks. Moreover, subgroup analysis showed that cancer risks in *GSTM1*-null was organ-specific, especially for lung cancer and nasopharyngeal cancer among smokers, and liver cancer, head and neck cancer and stomach cancer among drinkers. In addition to smoking, the pathogenesis of lung cancer was affected by various factors such as living habits, environment, genetics, etc. The results reflected in this study showed that the risk of lung cancer was higher among nonsmokers (OR = 1.699, *P < *.001) than that among smokers (OR = 1.542, *P < *.001). Consistent with cumulative evidence, we also found that tobacco was not a contributing factor to stomach^[52]^; cervical^[46,111,112]^; and bladder^[113]^ cancers. In studies of ethnic differences in *GSTM1* gene polymorphisms, The evidence showed that the frequency of *GSTM1*-null in Asians was higher than that in Caucasians and the interaction of *GSTM1*-null and smoking increased the risk of oral cancer in Asians than Caucasians.^[114,115]^ Our study showed that the overall cancer risks in *GSTM1*-null carriers were 1.515-fold among Asian and 1.312-fold among Caucasian smokers, respectively, suggesting that cancer risks related to *GSTM1*-null might be slightly higher in Asian smokers. But the interaction of *GSTM1*-null and drinking might increase cancer risks in Caucasian drinkers. In conclusion, *GSTM1*-null was associated with the overall increased cancer risks, especially in the lung, the bladder, the nasopharyngeal, the stomach and the cervical. And alcohol consumption might be a synergistic factor in promoting cancer risks of the liver, the head and neck and the stomach among *GSTM1*-null carriers.

For *GSTT1*, one study in 2013 indicated that *GSTT1* polymorphism might promote cancer development, especially in smokers.^[104]^ But for cancer of different organs, many scholars put forward specific views. Du et al^[106]^ found that smoking could rise the risk of esophageal cancer; Lao et al^[107]^ found that drinking played a critical role in promoting the development of gastric cancer. The outcome in this study found that *GSTT1*-null combined with smoking or drinking could significantly increase the overall cancer risks (OR = 1.356 for smoking; OR = 1.423 for drinking). *GSTT1*-null combined with smoking might be obviously related to the pathogenesis of prostate cancer and pancreatic cancer for the risk being as high as 2.847 times and 3.749 times, respectively. Although Zeng et al^[116]^ in 2016 found that alcohol consumption was not a co-factor of *GSTT1*-null in the development of gastric cancer, our findings suggested that an increased cancer risks might be associated with smoking (OR = 1.944, *P = *.045) and drinking (OR = 1.877, *P* = .021) among *GSTT1* -null carriers. In addition, the risk of cancers increased by 1.318-fold and1.543-fold among Caucasian smokers and drinkers, and by 1.701-fold and 2.250-fold among Asian smokers and drinkers, respectively. In conclusion, smoking and drinking were both synergistic factors in promoting the overall cancer risks among *GSTT1* -null carriers.

For *GSTP1rs1695*, cumulative researches confirmed that no statistical significance was found between *GSTP1* gene polymorphism and cancer risks.^[117–119]^ However, we noticed that Bao et al^[120]^ stated that the interaction of *GSTP1* gene mutation and smoking might reduce the risk of gastric cancer; and a huge review of Caucasian had estimated that smoking could modify lung cancer risks of *GSTP1* gene mutation in Caucasians.^[121]^ In this study, we observed that the *GSTP1* (AG + GG) variations might act as a protective factor among nondrinkers (OR = .840, *P = *.032). In addition, the risk of head and neck cancer also decreased among smokers. Nevertheless, *GSTP1rs1695* genetic variants might be negatively associated with cancer risks in Caucasian smokers or alcohol drinkers. However, due to the relatively small sample data, these conclusions should be treated with caution.

The limitations of this study were: The interactions between GSTs and other possible isozymes on cancer risks were not considered in this analysis. Cancers were caused by multiple factors; therefore, the synergistic or antagonistic effect of other factors should be considered as well as possible. Cancer risks might be more pronounced if the effects of genetic polymorphisms, alcohol and tobacco were combined together.^[16,40]^ GSTs might have overlapping affinities, and that the combined effects of detoxification functions in *GSTM1* and *GSTT1* may have a greater impact on disease than the effect of independent one.^[122]^ However, in the process of data extraction, some valuable articles could not be included in the analysis because of a lack of original data provided, resulting in the small sample size of data, especially for the analysis of the association between GSTs and cancers among drinking population. So, the sample data in this study was not available to further explore the relationship between the combined effects of gene-gene associations and smoking-drinking associations on cancers. Otherwise, the representativeness might be stronger to further explore the interaction between *GSTM1, GSTT1* and *GSTP1* polymorphisms and cancers among drinkers.

In conclusion, *GSTM1*-null might increase the risk of cancers, whether alone or in combination with smoking, but smoking and drinking might significantly contribute to increased cancer risks associated with *GSTT1*-null. No synergistic effect on the increased cancer risks was found between *GSTP1rs1695* and smoking or drinking. And the role of GSTs polymorphisms in combination with smoking or drinking on cancers might be influenced by ethnicities. Broader sample data and appropriate experimental design were needed to further investigate the effects of *GSTM1, GSTT1* and *GSTP1* polymorphisms on cancer among smokers or drinkers.

## Author contributions

**Conceptualization:** Cuiping Li.

**Investigation:** Qiurui Hu, Yonghui Huang, Zhenxia Wei.

**Validation:** Li Chen, Ying Luo.

**Writing – original draft:** Qiurui Hu.

**Writing – review & editing:** Cuiping Li, Xiaojie Li.
